# Human embryonic stem cell–derived mesenchymal stem cells ameliorate mitochondrial oxidative dysfunction in metabolic dysfunction–associated steatotic liver disease

**DOI:** 10.1093/stcltm/szag089

**Published:** 2026-10-30

**Authors:** Min Kyung Park, Yun Bin Lee, Se-Mi Jung, Yoo-Wook Kwon, Jung-Hwan Yoon

**Affiliations:** Department of Internal Medicine and Liver Research Institute, Seoul National University Hospital, Seoul National University College of Medicine, Seoul 03080, Republic of Korea; Department of Internal Medicine, Seoul National University Bundang Hospital, Seoul National University College of Medicine, Seongnam 13620, Republic of Korea; Department of Internal Medicine and Liver Research Institute, Seoul National University Hospital, Seoul National University College of Medicine, Seoul 03080, Republic of Korea; Department of Internal Medicine and Liver Research Institute, Seoul National University Hospital, Seoul National University College of Medicine, Seoul 03080, Republic of Korea; Biomedical Research Institute, Seoul National University Hospital, Seoul National University College of Medicine, Seoul 03080, Republic of Korea; Department of Internal Medicine and Liver Research Institute, Seoul National University Hospital, Seoul National University College of Medicine, Seoul 03080, Republic of Korea

**Keywords:** metabolic dysfunction–associated steatotic liver disease, mitochondria, embryonic stem cell, mesenchymal stem cell transplantation

## Abstract

**Introduction:**

Metabolic dysfunction-associated steatotic liver disease (MASLD) is a common chronic liver disease with significant clinical burden. This study investigated the therapeutic potential of embryonic stem cell-derived mesenchymal stem cells (ESC-MSCs) in cellular and murine models of MASLD.

**Methods:**

HepG2 cells were treated with palmitic acid (PA) and then co-cultured with ESC-MSCs. C57BL/6 mice were fed a choline-deficient, L-amino acid-defined, high-fat diet (CDAHFD) or a standard diet for 24 weeks. Either ESC-MSCs or vehicles were administered via tail vein injection at week 20. Lipid accumulation was assessed using Oil red O staining. Mitochondrial status was assessed using MitoTracker Red fluorescence, mtDNA content, reactive oxygen species (ROS), and antioxidant parameters.

**Results:**

ESC-MSC co-culture reduced PA-induced lipid accumulation, restored mitochondrial activity, decreased ROS, and increased superoxide dismutase (SOD) activity and glutathione redox balance. Expression of phosphorylated AMPK (p-AMPK), PGC1α, and PPARα was significantly upregulated. In CDAHFD-fed mice, ESC-MSC transplantation attenuated hepatic steatosis, restored mitochondrial DNA content, and reduced ROS accumulation with increased SOD activity and glutathione ratio. Hepatic expression of p-AMPK, PGC1α, and PPARα was also enhanced. These effects were abrogated by Compound C, an AMPK inhibitor. ESC-MSCs secreted hepatocyte growth factor (HGF), which activated AMPK, suggesting an HGF–AMPK axis may serve as a potential paracrine mechanism.

**Conclusion:**

ESC-MSCs ameliorated hepatic steatosis in MASLD models by restoring mitochondrial activity and reducing oxidative stress, possibly involving HGF-associated AMPK pathway modulation. These findings support the potential of ESC-MSC therapy for MASLD.

## Graphical Abstract

**Graphical Abstract:**
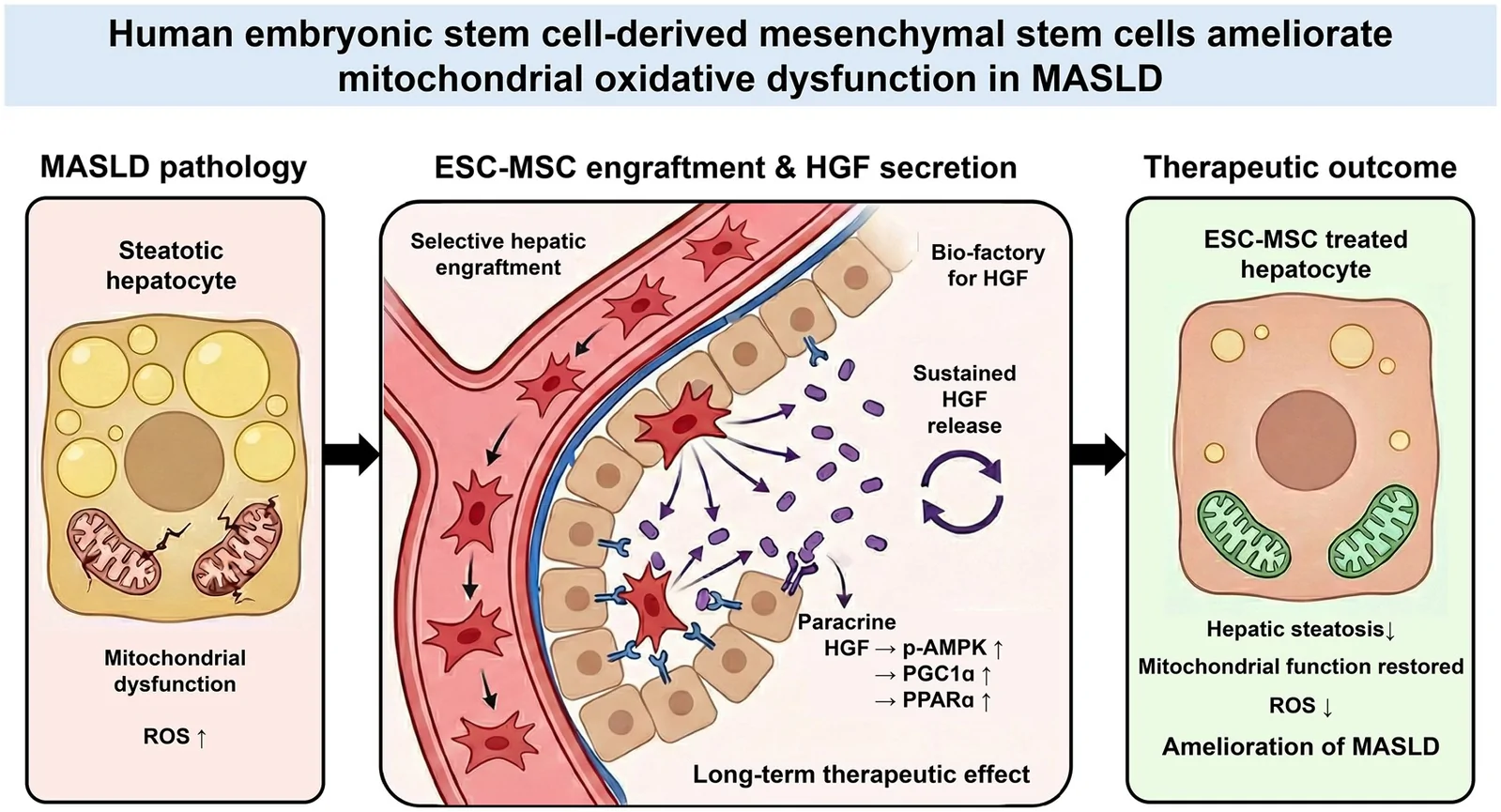

Significant statement This study demonstrates the therapeutic potential of specialized stem cells for treating metabolic dysfunction-associated steatotic liver disease (MASLD). Using cellular and animal models, we show that these cells were detected in liver tissue at the terminal endpoint, while restoring vital energy production and reducing harmful oxidative stress. Our findings identify a specific growth factor secreted by these cells as the key driver for reversing fat accumulation and improving metabolic function. By outlining this novel biological pathway, this research supports the development of scalable and targeted cell-based regenerative therapies as a promising approach for managing MASLD.

## Introduction

Metabolic dysfunction-associated steatotic liver disease (MASLD) is one of the most common chronic liver diseases, with an increasing prevalence exceeding 30%, resulting in a high disease burden worldwide.^1–3^ MASLD is associated with the risks of progression to liver cirrhosis and the development of malignancy, including hepatocellular carcinoma, leading to increased mortality.^2^^,^^4^ Despite these serious clinical outcomes, the pathophysiology of MASLD remains to be elucidated because complicated mechanisms are involved.

Several hypotheses have proposed a “multiple parallel hits” model, in which multiple simultaneous insults—such as insulin resistance, lipotoxicity, dysbiosis, and mitochondrial dysfunction—contribute to hepatic steatosis and inflammation.^5^ In particular, mitochondrial dysfunction and the resultant oxidative stress have been considered key drivers of MASLD progression.^6–8^ Alterations in mitochondrial structure and function, including reduced respiratory chain activity and increased reactive oxygen species (ROS), have been well-documented in patients with MASLD.^8^^,^^9^ These observations have spurred interest in mitochondrial pathways as therapeutic targets, even though there are no widely approved drugs yet.^8^^,^^10^^,^^11^

Ongoing advances in pharmacologic therapy have yielded promising candidates. Notably, resmetirom, a thyroid hormone receptor-β agonist, was approved in 2024 as the first pharmacotherapy for MASLD. Other agents targeting inflammation, lipid metabolism, and mitochondrial function—such as Aramchol, pioglitazone, and glucagon-like peptide-1 receptor agonists—are also under active investigation.^12^ While these agents show beneficial effects, they are limited by variable patient responses, short half-lives, and potential off-target effects.

Mesenchymal stem cells (MSCs), which are characterized by multi-lineage differentiation potential, low-immunogenicity, and homing effects, are used in various cell-based therapies.^13^^,^^14^ MSCs, which possess immune-modulatory and anti-inflammatory properties, are considered promising for the treatment of chronic liver diseases.^15–20^ Embryonic stem cell-derived mesenchymal stem cells (ESC-MSCs) are characterized by powerful proliferative capacity and immune privilege, making them suitable for clinical applications.^21^ In a previous study, human embryonic stem cells were successfully differentiated into ESC-MSCs, which could be expanded in vitro.^22^ This study aimed to investigate the therapeutic potential of ESC-MSCs on hepatic steatosis and mitochondrial dysfunction using cellular and murine models of MASLD.

## Methods

### Ethical statement

This study was approved by the Institutional Animal Care and Use Committee of Seoul National University Hospital (IACUC No. 20-0243-S1A0(2)). Animals were maintained in an AAALAC International-accredited facility (accreditation No. 001169) in accordance with Guide for the Care and Use of Laboratory Animals, 8th edition, NRC (2010). ESC-MSCs were derived from human embryonic stem cell lines as described previously,^22^ and the use of the ESC-MSCs was approved by the Institutional Review Board of Seoul National University Hospital (IRB No.: H-1410-093-619).

### Culture and preparation of ESC-MSCs

ESC-MSCs were generated from human embryonic stem cells (SNUhES3) via embryoid body formation and gelatin-based differentiation, as previously described.^22^ Cells expressed MSC-specific markers and multilineage differentiation capacity.^22^ ESC-MSCs were cultured in mesenchymal stem cell growth medium (PromoCell, Germany) at 37 °C.

### Development of MASLD cellular model and co-culture with ESC-MSCs

HepG2 cells (4 × 10^5^ cells/well) were seeded in a 6-well plate and treated with 100 μm palmitic acid (PA; Sigma-Aldrich, St. Louis, MO, USA) after serum starvation. After 16 hours of PA exposure, cells were co-cultured with 8 × 10^4^ ESC-MSCs (11–13 passages) per dish in a transwell insert (8.0 μm pore; BD Falcon, San Jose, CA, USA) for 18 hours (Figure S1A, see online supplementary material for a color version of this figure).

### Development of MASLD murine model and transplantation of ESC-MSCs

A choline-deficient, L-amino acid-defined, high-fat diet (CDAHFD) has been previously validated to effectively induce hepatic steatosis, fibrosis, and even tumorigenesis in long-term feeding models.^23–25^ Four-week-old male C57BL/6 mice (Orient Bio Inc., Seongnam, Korea) were used in this study. Standard chow mice served as a baseline control (*n* = 3), whereas mice fed the CDAHFD were randomly assigned to the vehicle (*n* = 5) or ESC-MSC (*n* = 7) groups prior to the initiation of treatment (Figure S1B, see online supplementary material for a color version of this figure). The mice had ad libitum access to food and autoclaved water. The control group received a standard diet containing 10 kcal% fat, 20 kcal% protein, and 70 kcal% carbohydrates (Champion Feed, Santiago, Chile), while the CDAHFD-fed groups were provided a CDAHFD, containing 60 kcal% fat, 0.1% methionine, and no added choline (Research Diets, New Brunswick, NJ, USA).^26^ ESC-MSCs (1 × 10^6^ cells, 9–10 passages) were intravenously injected via the tail vein into CDAHFD + ESC-MSC group mice at week 20, and the animals were sacrificed at week 24 for sample collection, by referring to regimens commonly used in previous preclinical liver injury studies.^27^

### Oil Red O staining and hepatic triglyceride measurement

Lipid accumulation in HepG2 cells and frozen liver sections was assessed by Oil Red O staining (Sigma-Aldrich, St Louis, MO, USA) and quantified by absorbance at 510 nm as previously described.^28^ Hepatic triglyceride (TG) levels were measured using a hepatic TG quantification assay kit (Abcam, Cambridge, UK), according to the manufacturer’s instructions.

### Mitochondrial staining assay and mtDNA quantification

Mitochondrial fluorescence was analyzed using MitoTracker Red FM (200 nM; Invitrogen, Carlsbad, CA, USA), followed by DAPI nuclear staining. Mitochondrial DNA (mtDNA) was quantified by qPCR using the QIAamp DNA Mini Kit (QIAGEN, Hilden, Germany) and StepOnePlus real-time PCR System (Applied Biosystems, Foster City, CA, USA) with GoTaq qPCR Master Mix (Promega, Madison, WI, USA) according to the manufacturers’ instructions. Primer sequences used for the quantification of human nuclear DNA (β-actin), human mtDNA, mouse nuclear DNA (β-globin), and mouse mtDNA are listed in Table S1.

### Measurement of oxidative stress parameters

Intracellular ROS levels were assessed using the OxiSelect ROS Assay Kit (Cell Biolabs, San Diego, CA, USA) based on 2′,7′-dichlorofluorescein diacetate, as previously described.^29^ Superoxide dismutase (SOD) activity was assessed using a colorimetric SOD assay kit (BioVision, Milpitas, CA, USA) by measuring absorbance at 450 nm after incubation with tetrazolium-based reagents. The intracellular ratio of reduced (GSH) to oxidized glutathione (GSSG) was determined using a GSH/GSSG assay kit (Abcam) based on the DTNB recycling method, with absorbance measured at 412 nm following deproteinization with sulfosalicylic acid.

### Quantitative real-time polymerase chain reaction

Total RNA was extract using Trizol (Invitrogen), and cDNA was synthesized from 2 µg RNA using the PrimeScript RT reagent Kit (TaKaRa, Shiga, Japan). qPCR was performed using GoTaq qPCR Master Mix and StepOnePlus system. Primer sequences are listed in Table S2; β-actin or 18S rRNA served as internal controls.

### Western blot analysis

Proteins were extracted with RIPA buffer, resolved by SDS-PAGE, and transferred to membranes. Membranes were incubated at 4 °C overnight with primary antibodies as follows: anti-AMP-activated protein kinase (AMPK; 1:2000; Cell Signaling Technology, Danvers, MA, USA), anti-Thr172-phosphorylated AMPK (1:1000; Cell Signaling Technology), anti-peroxisome proliferator-activated receptor gamma coactivator 1-alpha (PGC1α; 1:1500; Abcam), anti-peroxisome proliferator-activated receptor alpha (PPARα; 1:2000; Invitrogen), anti-glyceraldehyde-3-phosphate dehydrogenase (GAPDH; 1:10 000; Thermo Fisher Scientific, Cleveland, OH, USA), and anti-α-tubulin (1:5000; Cell Signaling Technology). Anti-rabbit IgG (1:10 000; Invitrogen) and anti-mouse IgG (1:10 000; Invitrogen) were used as secondary antibodies.

### Evaluation of AMPK dependency using Compound C treatment

To evaluate AMPK dependency, HepG2 cells were subjected to serum starvation for 5 hours and then pretreated with Compound C (10 μm; Selleckchem, Houston, TX, USA), an AMPK inhibitor, for 1 hour prior to PA exposure (100 μm, 16 hours). Compound C was continuously maintained in the culture medium throughout the subsequent 18-hour co-culture with ESC-MSCs until cell harvest. Lipid accumulation, gene and protein expression (p-AMPK, PGC1α, and PPARα), and oxidative stress parameters (ROS levels, SOD activity, and the GSH/GSSG ratio) were assessed as described above.

### Blood chemistry

Serum levels of aspartate aminotransferase (AST) and alanine aminotransferase (ALT) were analyzed using an automatic biochemical analyzer (HITACHI 7180, Tokyo, Japan) and expressed in IU/L.

### Hepatocyte growth factor (HGF) assays and histological analysis

Details of HGF quantification, HGF treatment assays, and histological evaluation, and human-specific Alu qPCR for ESC-MSC tissue retention and biodistribution are provided in the Supplementary Methods.

### Statistical analysis

Data are presented as the mean ± standard deviation. Group differences were evaluated by one-way analysis of variance followed by Tukey’s post-hoc test (for 3 groups) or Fisher’s least significant difference test (for 6-group Compound C assays). A *P*-value less than .05 was considered statistically significant. Statistical analyses were performed using GraphPad Prism version 9.4.1 (GraphPad Software, San Diego, CA, USA). For visual presentation, Gemini (Google LLC, version 2.5 Flash, accessed on December 10, 2025) was used to assist in drafting the graphical abstract, and the authors have verified the final content for accuracy and integrity. Detailed datasets are available from the corresponding author upon reasonable request.

## Results

### ESC-MSCs attenuate lipid accumulation and oxidative stress in the MASLD cellular model

PA-treated HepG2 cells exhibited significant intracellular lipid accumulation, which was markedly reduced by indirect co-culture with ESC-MSCs (*P *< .001; Figure 1A), supporting the establishment of a MASLD model and the therapeutic potential of ESC-MSCs.

**Figure 1.**
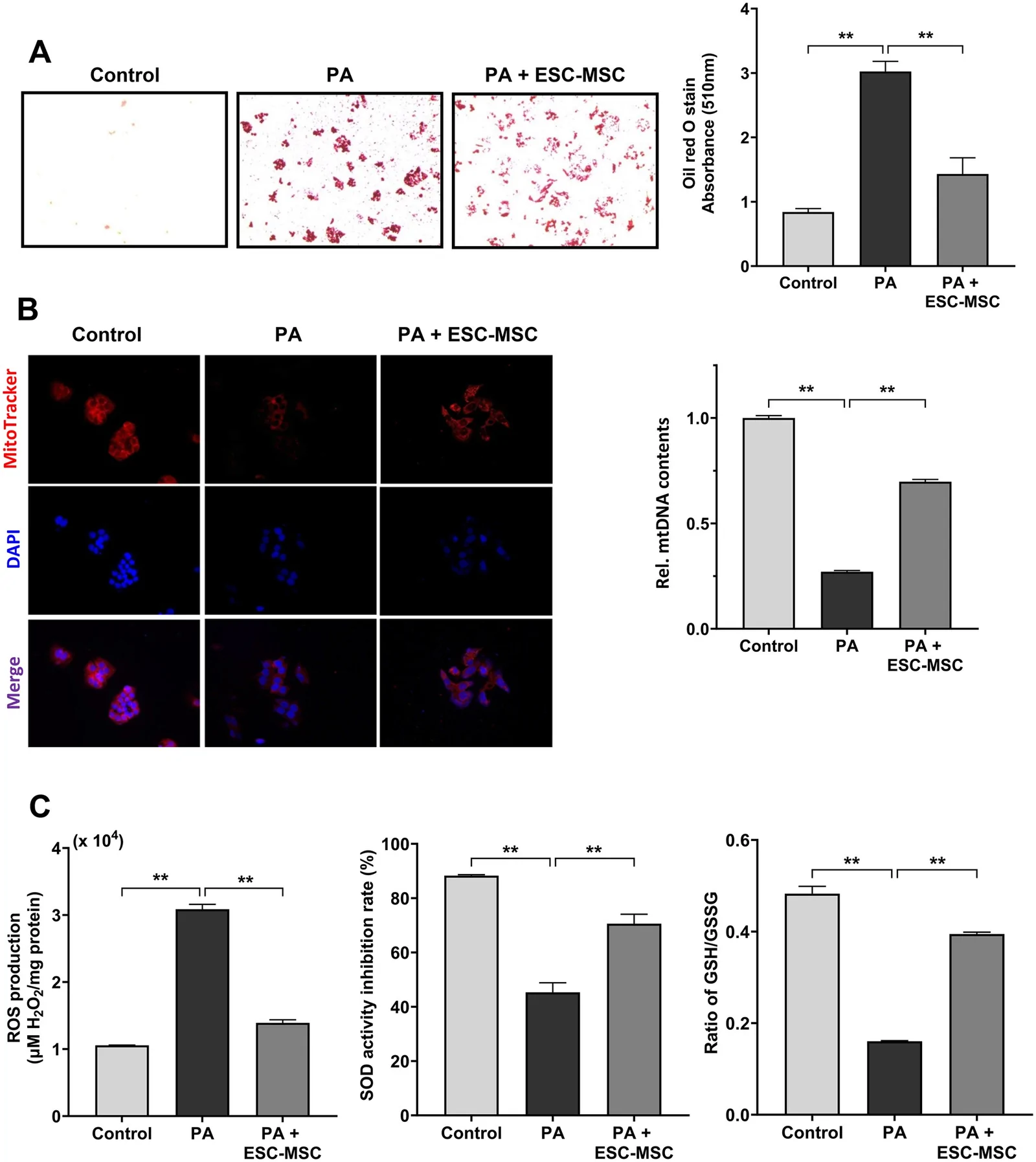
ESC-MSCs attenuate lipid accumulation and restore mitochondrial oxidative stress in PA-treated HepG2 cells. (A) Representative Oil Red O staining images showing intracellular lipid accumulation in control, PA-treated, and PA + ESC-MSC co-cultured HepG2 cells. (B) Quantification of MitoTracker Red fluorescence and mitochondrial DNA content. (C) Measurement of intracellular ROS, SOD activity, and the GSH/GSSG ratio. Data are presented as mean ± SD; Group differences were evaluated by one-way ANOVA followed by Tukey’s post-hoc test; **P < 0.01. Abbreviations: ESC-MSC, embryonic stem cell–derived mesenchymal stem cell; GSH, reduced glutathione; GSSG, oxidized glutathione; mtDNA, mitochondrial DNA; PA, palmitic acid; ROS, reactive oxygen species; SOD, superoxide dismutase.

Furthermore, PA exposure significantly decreased mitochondrial signal (MitoTracker Red) and mtDNA content (*P *< .001; Figure 1B), while increasing intracellular ROS levels and impairing antioxidant defenses (*P *< .001; Figure 1C), including reductions in SOD activity (*P *= .001; Figure 1C), and GSH/GSSG ratio (*P *< .001; Figure 1C). ESC-MSC co-culture effectively restored mitochondrial signal and mtDNA content (*P *< .001; Figure 1B), reduced ROS production (*P *< .001; Figure 1C), and normalized antioxidant activities to near-control levels (*P *= .006; Figure 1C), indicating that ESC-MSCs may ameliorate lipid accumulation and mitochondrial oxidative stress in the MASLD cellular model.

### ESC-MSCs activate the AMPK–PGC1α–PPARα axis in the MASLD cellular model

p-AMPK (Thr172), a key regulator of lipid metabolism and mitochondrial biogenesis,^30–32^ was reduced by PA treatment (*P *= .03; Figure 2A) but significantly increased following ESC-MSC co-culture (*P *< .001; Figure 2A). PGC1α, a downstream target of p-AMPK, has emerged as a pivotal regulator of mitochondrial dynamics and oxidative function.^33^ ESC-MSCs also restored the expression of PGC1α at both mRNA (*P *< .001; Figure 2B) and protein levels (*P *= .03; Figure 2B) and increased PPARα expression, which was suppressed by PA treatment (*P *< .001; Figure 2C). Collectively, these findings suggest that ESC-MSC co-culture restores the AMPK–PGC1α–PPARα signaling axis in the MASLD cellular model.

**Figure 2.**
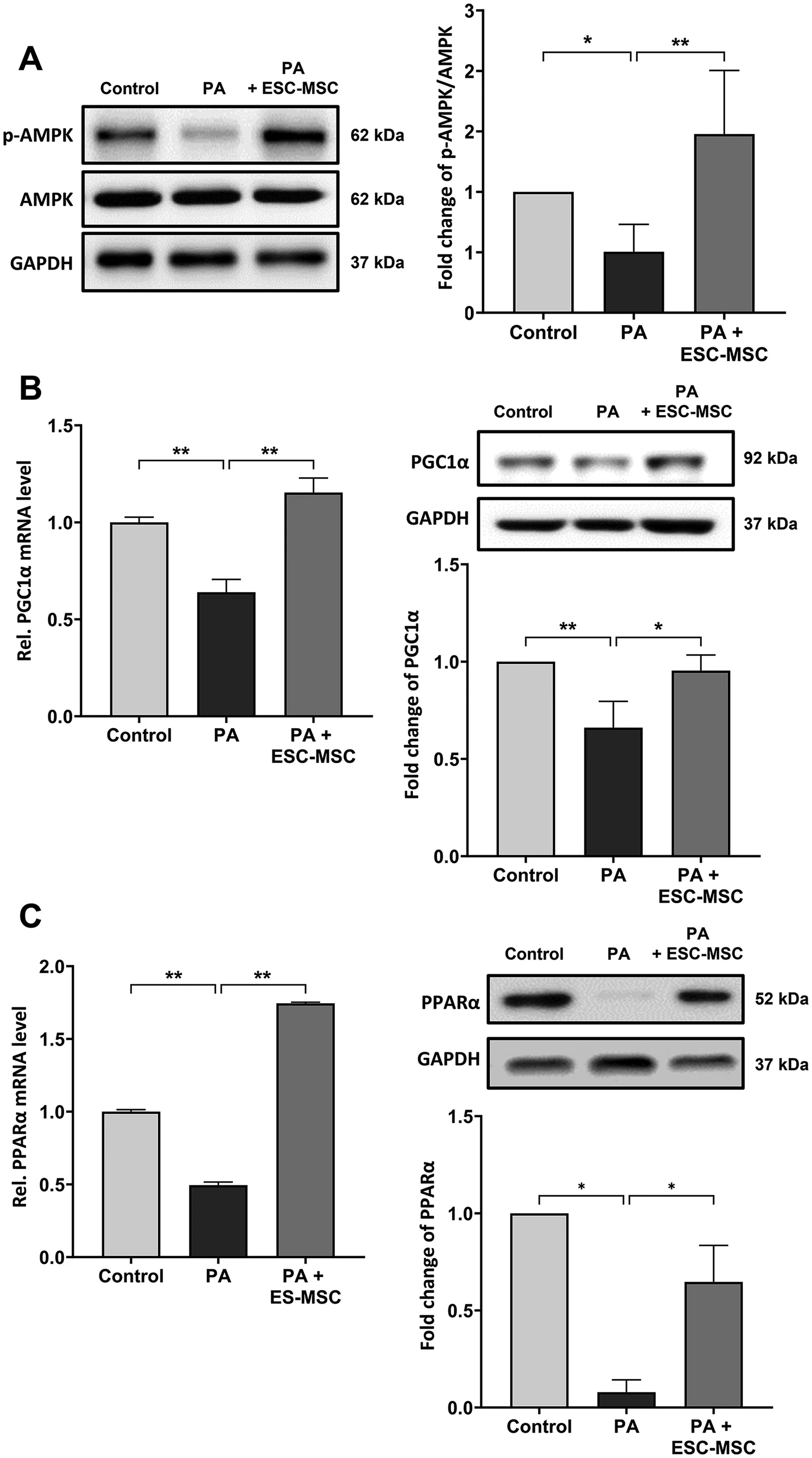
ESC-MSCs activate the AMPK–PGC1α–PPARα signaling axis in the MASLD cellular model. (A) Representative Western blots and quantification of p-AMPK and total AMPK. (B) Expression of PGC1α at the mRNA and protein levels. (C) Expression of PPARα at the mRNA and protein levels in control, PA, and PA + ESC-MSC groups. GAPDH was used as the loading control. Data are expressed as mean ± SD; Group differences were evaluated by one-way analysis of variance followed by Tukey’s post-hoc test; **P < .01. Abbreviations: AMPK, AMP-activated protein kinase; ESC-MSC, embryonic stem cell–derived mesenchymal stem cell; GAPDH, glyceraldehyde-3-phosphate dehydrogenase; MASLD, metabolic dysfunction–associated steatotic liver disease; PA, palmitic acid; PGC1α, peroxisome proliferator–activated receptor gamma coactivator 1-alpha; PPARα, peroxisome proliferator–activated receptor alpha; qPCR, quantitative polymerase chain reaction.

### ESC-MSCs localize in murine liver tissue and ameliorate hepatic steatosis with restoration of mitochondrial oxidative balance

Immunohistochemical staining for human HLA revealed positive cells in the liver of CDAHFD + ESC-MSC mice, but not in control or CDAHFD + vehicle groups, nor in lung tissue (Figure S2A and B, see online supplementary material for a color version of this figure). To further validate this finding quantitatively, human-specific Alu qPCR was performed on genomic DNA extracted from liver, lung, and spleen tissues. Quantitative human-specific Alu qPCR revealed that a distinct human cell signal was detected in liver tissue of the CDAHFD + ESC-MSC group (0.148 ± 0.019 cell equivalents/reaction), whereas all control livers, lungs, and spleens remained below the quantification threshold (0.1 cell equivalents/reaction; Figure S2C, see online supplementary material for a color version of this figure). This localization coincided with significant histological and biochemical improvements. CDAHFD-fed mice exhibited hepatic macrovesicular steatosis (*P *< .001; Figure 3A), along with elevated hepatic triglyceride (TG) levels (*P *= .004; Figure 3B) and increased serum levels of AST (*P *< .001; Figure 3B) and ALT (*P *= .04; Figure 3B) compared with control mice. ESC-MSC transplantation significantly attenuated macrovesicular steatosis (*P *< .001; Figure 3A) and serum AST levels (*P *< .001; Figure 3B), accompanied by numerical reductions in hepatic TG (*P *= .10; Figure 3B) and serum ALT levels (*P *= .07; Figure 3B).

**Figure 3.**
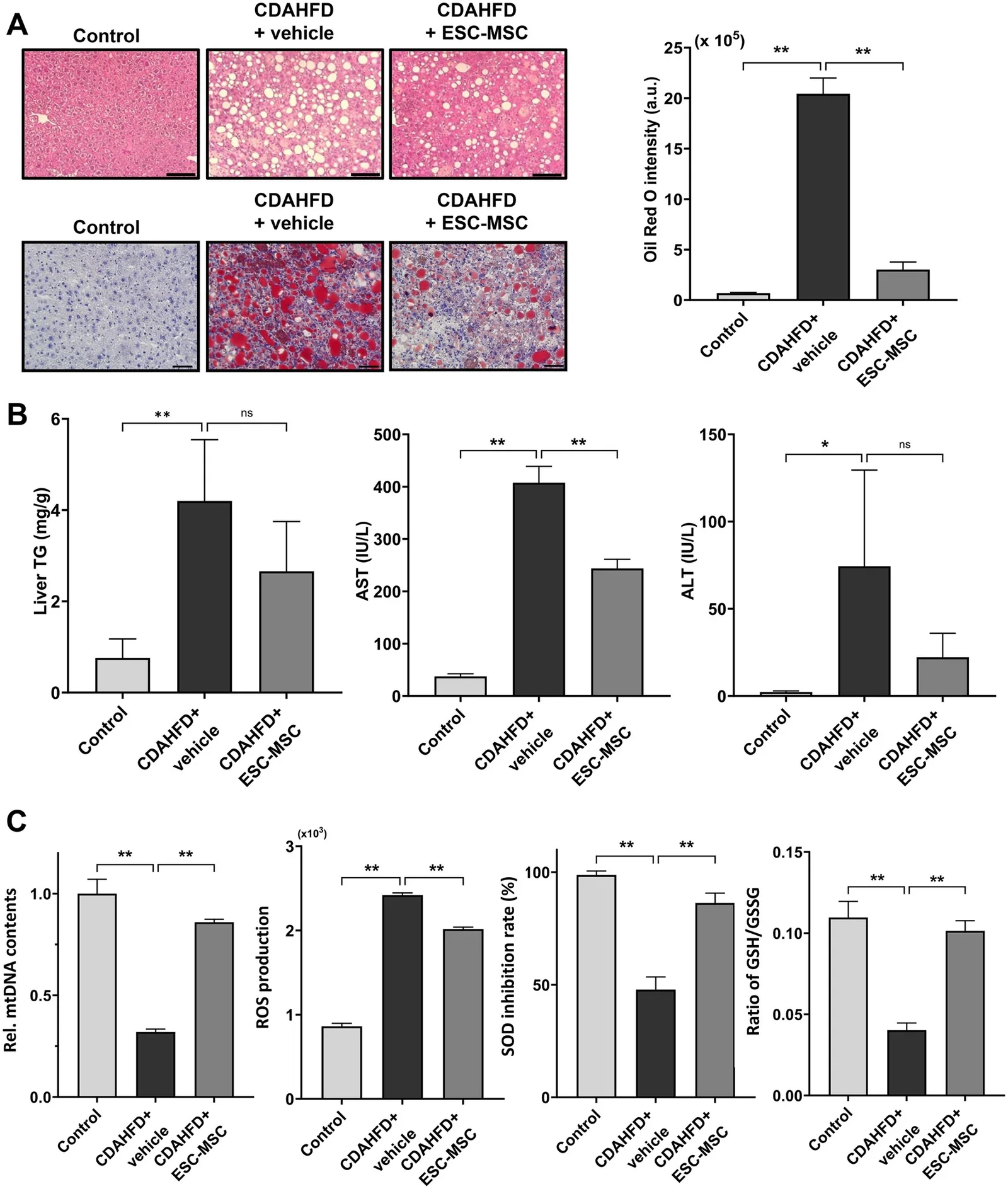
ESC-MSC transplantation ameliorates hepatic steatosis and restores mitochondrial oxidative balance in CDAHFD-fed mice. (A) Representative H&E and Oil Red O–stained liver sections and quantitative analysis of hepatic lipid accumulation in control, CDAHFD + vehicle, and CDAHFD + ESC-MSC groups. (B) Quantitative analysis of liver TG, serum levels of AST and ALT. (C) Mitochondrial DNA content, ROS levels, SOD activity, and GSH/GSSG ratio. Data are shown as mean ± SD; group differences were evaluated by one-way analysis of variance followed by Tukey’s post-hoc test; *P < .05, **P < .01. Abbreviations: ALT, alanine aminotransferase; AST, aspartate aminotransferase; CDAHFD, choline-deficient, L-amino acid-defined, high-fat diet; ESC-MSC, embryonic stem cell–derived mesenchymal stem cell; GSH, reduced glutathione; GSSG, oxidized glutathione; H&E, hematoxylin and eosin; mtDNA, mitochondrial DNA; ROS, reactive oxygen species; SOD, superoxide dismutase; TG, triglyceride.

Moreover, ESC-MSCs restored hepatic mtDNA content (*P *= .002; Figure 3C), reduced ROS accumulation (*P *= .002; Figure 3C), and normalized SOD activity (*P *= .006; Figure 3C) and the GSH/GSSG ratio (*P *= .007; Figure 3C), suggesting a therapeutic impact on hepatic steatosis and mitochondrial oxidative stress in vivo. Furthermore, ESC-MSC administration significantly attenuated CDAHFD-induced hepatic upregulation of pro-inflammatory cytokines (*Ifng*, *Tnf*, and *Il6*) without exacerbating local inflammatory responses (Figure S3, see online supplementary material for a color version of this figure), indicating no overt aggravation of local hepatic inflammation at the terminal endpoint.

### ESC-MSCs enhance AMPK–PGC1α–PPARα signaling in the MASLD murine model

In the present study, p-AMPK levels were significantly increased in the CDAHFD + vehicle group compared to the control group (*P *= .02; Figure 4A). Notably, ESC-MSC transplantation further elevated p-AMPK levels relative to the CDAHFD + vehicle group (*P *= .04; Figure 4A), suggesting additive activation of this energy-sensing kinase. Both mRNA and protein levels of PGC1α were increased in the CDAHFD + vehicle group and were further elevated by ESC-MSC transplantation (*P *= .02; Figure 4B). In contrast, the expression of PPARα—transcriptionally regulated by PGC1α and essential for fatty acid oxidation—was paradoxically decreased in the CDAHFD + vehicle group despite PGC1α activation (*P *< .001; Figure 4C). This discrepancy suggests additional regulatory mechanisms affecting PPARα, such as microRNA-21, which has been shown to suppress PPARα expression in methionine-choline-deficient diet models similar to CDAHFD.^34^ Importantly, ESC-MSC transplantation reversed PPARα suppression, significantly increasing both its mRNA and protein expression (*P *= .009; Figure 4C) and thereby restoring the AMPK–PGC1α–PPARα signaling axis. Collectively, these findings indicate that while CDAHFD alone partially activates AMPK and PGC1α, ESC-MSC transplantation may further enhance this pathway and restore downstream targets such as PPARα, thereby improving hepatic metabolism and mitochondrial oxidative stress in MASLD.

**Figure 4.**
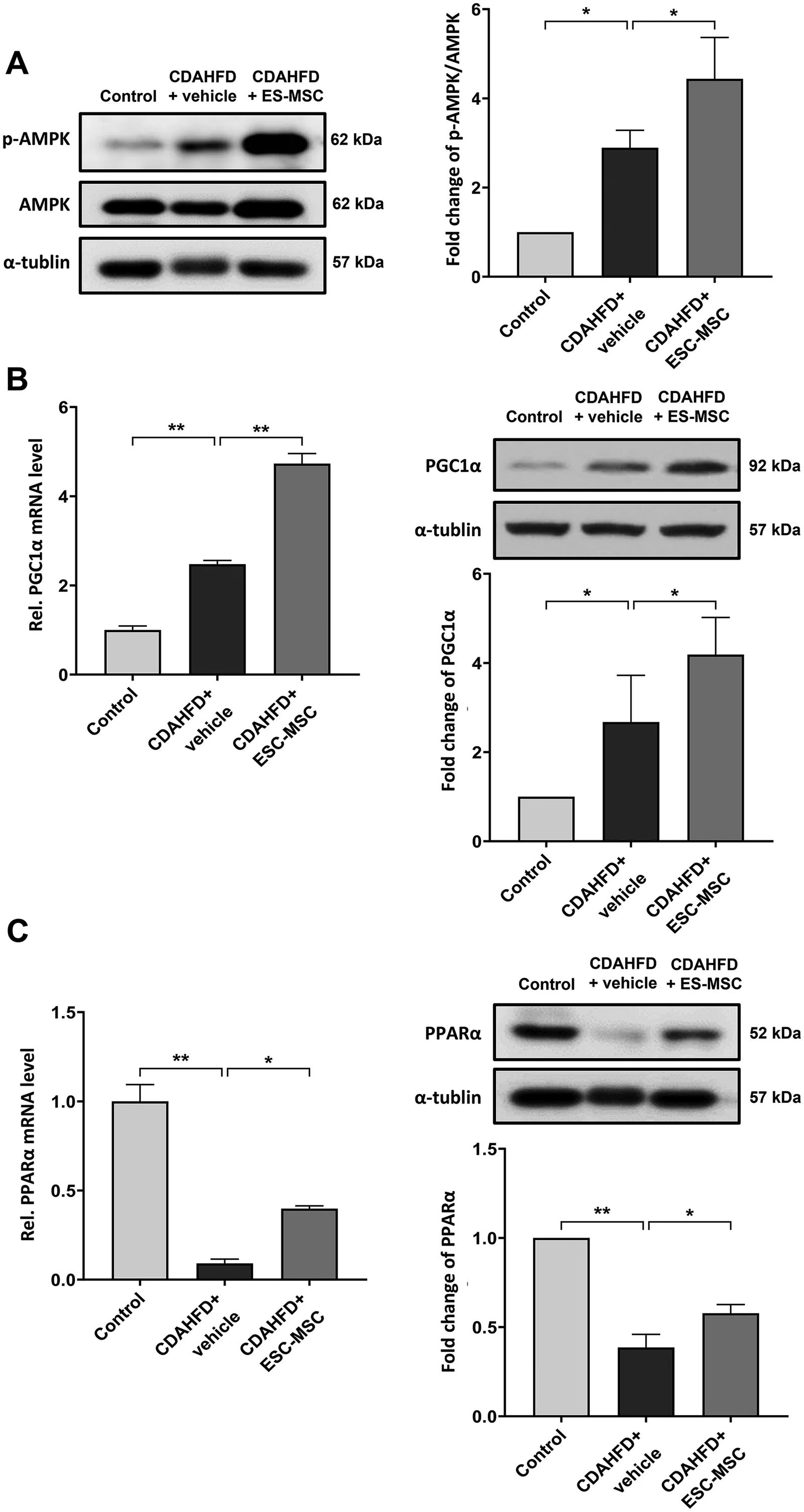
ESC-MSCs enhance AMPK–PGC1α–PPARα signaling in the MASLD murine model. (A) Representative Western blots and quantification of p-AMPK and total AMPK. (B) Expression of PGC1α at the mRNA and protein levels. (C) Expression of PPARα in control, CDAHFD + vehicle, and CDAHFD + ESC-MSC mice. α-Tubulin was used as a loading control. Data are presented as mean ± SD; group differences were evaluated by one-way analysis of variance followed by Tukey’s post-hoc test; *P < .05, **P < .01. Abbreviations: AMPK, AMP-activated protein kinase; CDAHFD, choline-deficient, L-amino acid-defined, high-fat diet; ESC-MSC, embryonic stem cell–derived mesenchymal stem cell; PGC1α, peroxisome proliferator–activated receptor gamma coactivator 1-alpha; PPARα, peroxisome proliferator–activated receptor alpha.

### AMPK inhibition attenuates the therapeutic effects of ESC-MSCs

To investigate the mechanisms underlying ESC-MSC action, we employed Compound C, an AMPK inhibitor. Pretreatment with Compound C significantly attenuated ESC-MSC–mediated improvements in lipid accumulation, as shown by Oil Red O staining (*P *< .001; Figure 5A). Furthermore, the ESC-MSC–induced upregulation of p-AMPK (*P *< .001; Figure 5B) and PGC1α was suppressed by Compound C (*P *= .007; Figure 5B). Compound C also abrogated the ESC-MSC–mediated restoration of mitochondrial oxidative function, including mtDNA content, ROS levels, SOD activity, and GSH/GSSG ratio (all *P *< .001; Figure 5C). These findings confirm that AMPK activation is essential for the therapeutic effects of ESC-MSCs.

**Figure 5.**
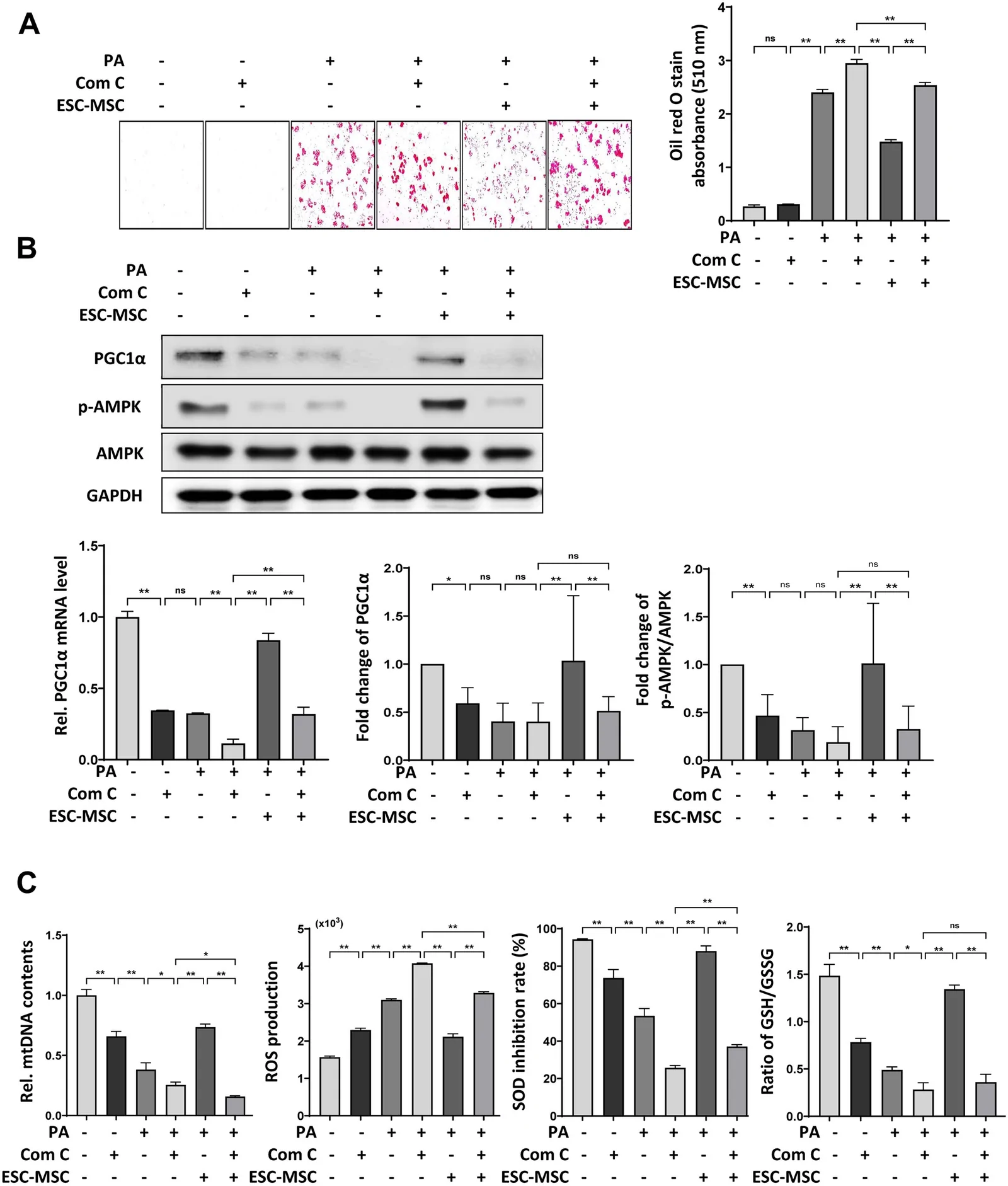
AMPK inhibition by compound C abrogates the therapeutic effects of ESC-MSCs. (A) Representative Oil Red O staining in control, PA, PA + ESC-MSC, and PA + ESC-MSC + Compound C groups. (B) Western blot and quantification of p-AMPK and PGC1α expression. (C) Quantitative analysis of mitochondrial DNA content, ROS levels, SOD activity, and GSH/GSSG ratio. Data are presented as mean ± SD; group differences were evaluated by one-way analysis of variance followed by Fisher’s least significant difference post-hoc test; *P < .05, **P < .01, ns, not significant. Abbreviations: AMPK, AMP-activated protein kinase; ESC-MSC, embryonic stem cell–derived mesenchymal stem cell; mtDNA, mitochondrial DNA; PA, palmitic acid; PGC1α, peroxisome proliferator–activated receptor gamma coactivator 1-alpha; ROS, reactive oxygen species; SOD, superoxide dismutase.

### Paracrine HGF as a candidate mediator associated with AMPK pathway activation

To identify upstream mediators of AMPK activation, HGF levels were measured in the co-culture supernatant and found to be significantly elevated under ESC-MSC co-culture conditions, consistent with prior evidence that hESC-derived MSCs secrete abundant HGF (*P *< .001; Figure S4A, see online supplementary material for a color version of this figure).^35^ Furthermore, to evaluate in vivo tissue presence, human-specific HGF ELISA was performed on liver lysates, detecting quantifiable human HGF in the CDAHFD + ESC-MSC group, whereas control and CDAHFD + vehicle groups were below the assay lower limit of quantification (Figure S4B, see online supplementary material for a color version of this figure). To further evaluate the role of HGF, recombinant human HGF was administered to PA-treated HepG2 cells, which led to increased phosphorylation of AMPK (*P *= .05; Figure S4C, see online supplementary material for a color version of this figure) and upregulation of PGC1α expression compared to PA treatment alone (*P *= .006; Figure S4D, see online supplementary material for a color version of this figure). These results suggest that ESC-MSC–derived HGF represents a plausible candidate paracrine mediator that may contribute to AMPK pathway modulation and highlight a paracrine mechanism through which ESC-MSCs may confer metabolic benefits associated with HGF signaling.

## Discussion

This study investigated the therapeutic effect of ESC-MSCs using both cellular and murine models of MASLD. Our models demonstrated consistent pathophysiologic features relevant to clinical MASLD, such as intracellular lipid accumulation, mitochondrial dysfunction, and elevated oxidative stress. Transplanted ESC-MSCs localized to the MASLD liver without detectable extrahepatic distribution, indicating targeted localization to the diseased liver. ESC-MSC co-culture or transplantation ameliorated hepatic steatosis, restored mitochondrial content and redox balance, and activated AMPK–PGC1α–PPARα signaling, indicating the broad therapeutic potential of ESC-MSCs.

ESC-MSCs represent a promising alternative to adult tissue-derived MSCs, which have shown therapeutic potential in both preclinical and clinical settings for patients with advanced chronic liver disease.^15^ While bone marrow- and adipose-derived MSCs have demonstrated efficacy in improving hepatic fibrosis and liver function, especially in alcoholic cirrhosis and metabolic dysfunction-associated steatohepatitis models, their clinical application is limited by challenges such as impractical autologous collection in elderly or cirrhotic patients and donor-related variability that affects reproducibility and scalability.^20^^,^^36^^,^^37^ To overcome these limitations, ESC-MSCs have emerged as a promising alternative candidate.^22^^,^^38^ They can be reliably derived from human embryonic stem cell lines, maintain stable proliferation even after multiple freeze–thaw cycles, and show no tumorigenic potential in immunodeficient mice. In our study, the ESC-MSC line exhibited a well-characterized profile with high expansion capacity, enabling repeated therapeutic applications.^22^ Notably, ESC-MSCs selectively localized to injured liver tissue rather than extrahepatic organs, thereby enhancing therapeutic efficacy and minimizing systemic side effects. These features make ESC-MSCs a uniform, scalable, and safe cell source for liver-targeted regenerative therapies, including those for chronic liver diseases such as MASLD.

The therapeutic benefit of ESC-MSCs appears to be mediated by restoration of mitochondrial flexibility, a function that is impaired as MASLD progresses from steatosis to steatohepatitis.^39^ In our models, PA treatment or CDAHFD feeding reduced mtDNA content and increased ROS, indicating loss of mitochondrial adaptive capacity. ESC-MSCs ameliorated these changes and restored oxidative function, highlighting their potential to halt or reverse mitochondrial dysfunction in MASLD. This is particularly relevant as mitochondrial dysfunction has been identified not only as a hallmark but also a potential driver of MASLD progression, influencing both lipid accumulation and inflammatory cascades.^7^^,^^40^

A key mechanistic insight from the present study is the activation of AMPK by ESC-MSCs, a central regulator of energy homeostasis, lipid metabolism, and mitochondrial biogenesis.^41^ AMPK regulation in MASLD varies depending on the dietary model. While AMPK activity is generally decreased in high-fat diet-fed models, paradoxical upregulation of p-AMPK has been observed in CDAHFD-fed models, possibly due to their unique metabolic features—such as lack of obesity, lower blood insulin levels, and reduced blood glucose levels.^23^ Under these choline-deficient conditions, elevated baseline p-AMPK likely represents a compensatory bioenergetic stress response to acute intrahepatic ATP depletion rather than physiological homeostasis. Importantly, this compensatory phosphorylation in vehicle-treated mice failed to sustain downstream metabolic cascades, as evidenced by repressed PGC1α and PPARα levels. Our study demonstrated that ESC-MSC treatment further enhanced AMPK pathway activation and functionally restored downstream PGC1α and PPARα expression, both in vitro and in vivo. The essential role of AMPK was confirmed through experiments using Compound C, which abolished the therapeutic effects of ESC-MSCs on lipid accumulation, redox balance, and gene expression. Pharmacological AMPK agonists such as AICAR have also demonstrated efficacy in MASLD models^42^; however, their effects are often transient and require frequent dosing. In contrast, ESC-MSCs exert a dual mechanism—sustained AMPK activation through paracrine HGF secretion and hepatic localization—that may confer longer-lasting and more targeted therapeutic effects. Additionally, ESC-MSCs showed no evidence of lung accumulation, underscoring their advantage in targeted delivery.

PGC1α, a critical transcriptional coactivator for mitochondrial function and lipid oxidation,^33^ was also upregulated by ESC-MSC treatment. Previous genetic studies have linked PGC1α polymorphisms with increased susceptibility to MASLD, highlighting its clinical relevance.^43^ However, the development of drugs that directly modulate PGC1α activity has been limited due to structural complexity and nuclear confinement. Therefore, targeting upstream regulators such as AMPK is a more feasible strategy to modulate PGC1α activity. Our study supports this concept, showing that ESC-MSCs indirectly activate PGC1α via AMPK stimulation. Further exploration of downstream targets, including NRFs, SIRT1, or autophagy-related regulators, may expand our understanding of how ESC-MSCs restore hepatic metabolic homeostasis.

This study has several strengths. First, this study integrates both in vitro and in vivo models, providing a consistent mechanistic link from cellular lipid metabolism to systemic liver pathology. Second, the identification of HGF as a paracrine activator of AMPK signaling reveals a key therapeutic axis in ESC-MSC-mediated protection. Moreover, the demonstration of hepatic localization over extrahepatic tissues highlights the potential for targeted and sustained therapeutic delivery.

Nonetheless, several limitations should be acknowledged. The use of a single ESC-MSC line may not fully capture the variability among different ESC-derived lines. Long-term safety evaluations, including fibrosis progression and tumorigenic potential in chronic settings, remain to be addressed. In addition, the therapeutic dose of ESC-MSCs was fixed based on prior studies using different stem cell sources, and time-course evaluations, dose-determining studies, and repeated-injection schedules are necessary to define the optimal therapeutic window and durability of treatment. Although human-specific Alu qPCR confirmed terminal hepatic retention, our single-endpoint design could not determine whether transplanted cells actively proliferate or persist long-term in vivo. Future studies utilizing serial tracking or proliferative markers (such as Ki-67 co-staining) are needed to clarify in vivo cell kinetics. Furthermore, while hepatic transcript levels showed no overt aggravation of hepatic expression of pro-inflammatory cytokine genes (*Ifng*, *Tnf*, and *Il6*), our analysis was restricted to a single terminal endpoint within liver tissue, and comprehensive evaluations of systemic immune responses and humoral xenoreactivity remain warranted. Additionally, while recombinant HGF activated AMPK in vitro and human HGF was detected in liver tissue lysates, our study did not include functional loss-of-function validations, such as HGF neutralization, c-MET receptor blockade, or HGF knockdown in ESC-MSCs; thus, whether HGF is an obligatory mediator among other secretome components warrants further investigation. Moreover, while MitoTracker Red staining and mtDNA copy number served as surrogate markers of mitochondrial activity and abundance, respectively, future studies incorporating direct bioenergetic assessments—such as oxygen consumption rate and ATP production—are warranted. Another consideration is the small control group (*n* = 3) and uneven sample allocation inherent to our proof-of-concept setup, warranting validation in larger, balanced cohorts. Finally, direct side-by-side functional comparisons with adult tissue-derived MSCs (such as bone marrow-derived or adipose-derived MSCs) remain to be performed to define relative therapeutic potency. Future directions should include the use of AMPK knockout models or siRNA-mediated gene silencing to robustly confirm the mechanistic involvement of AMPK in ESC-MSC–mediated effects. In parallel, long-term studies assessing safety, tumorigenicity, and fibrosis progression in chronic liver injury models are essential for clinical translation.

In conclusion, ESC-MSCs ameliorated hepatic steatosis in both cellular and MASLD murine models through attenuation of mitochondrial oxidative stress and activation of AMPK–PGC1α–PPARα signaling. Their hepatic localization and HGF secretion may contribute to sustained metabolic improvements compared with transient pharmacological interventions. Considering their regenerative capacity, immunomodulatory properties, and scalability, ESC-MSCs may represent a promising therapeutic approach for metabolic liver disease.

## Supplementary Material

szag089_Supplementary_Data

## Data Availability

The data underlying this article will be shared on reasonable request to the corresponding author.
