# Supplementary material for: Human embryonic stem cell–derived mesenchymal stem cells ameliorate mitochondrial oxidative dysfunction in metabolic dysfunction–associated steatotic liver disease
Source: Stem Cells Transl Med. 2026 Oct 30;15(11):szag089. doi: 10.1093/stcltm/szag089 (PMC13719285; doi:10.1093/stcltm/szag089)
Supplement: Supplementary file 1 [file szag089_supplementary_data.zip › Supplementary Materials_Park et al_revised_clean_v2.docx]

[Supplementary Information]

**Human embryonic stem cell-derived mesenchymal stem cells ameliorate mitochondrial oxidative dysfunction in MASLD**

**Running title:** hESC-MSCs improve mitochondrial function in MASLD

**Authors:** Min Kyung Park^1,2^, Yun Bin Lee^1*^, Se-Mi Jung^1^, Yoo-Wook Kwon^3*^, Jung-Hwan Yoon^1^

^1^*Department of Internal Medicine and Liver Research Institute, Seoul National University Hospital, Seoul National University College of Medicine, Seoul, Republic of Korea*

^2^*Department of Internal Medicine, Seoul National University Bundang Hospital, Seoul National University College of Medicine, Seongnam, Gyeonggi, Republic of Korea*

^3^*Biomedical Research Institute, Seoul National University Hospital, Seoul, Republic of Korea*

* These two authors contributed as co-corresponding authors.

**Address all correspondence and requests for reprints to:**

Yun Bin Lee, MD, PhD

Professor

Department of Internal Medicine and Liver Research Institute

Seoul National University Hospital

Seoul National University College of Medicine

101 Daehak-ro, Jongno-gu,

Seoul 03080, Republic of Korea

E-mail: yblee@snu.ac.kr

Tel: 82-2-2072-2228, Fax: 82-2-743-6701

or

Yoo-Wook Kwon, PhD

Professor

Biomedical Research Institute

Seoul National University Hospital

Seoul National University College of Medicine

101 Daehak-ro, Jongno-gu,

Seoul 03080, Republic of Korea

E-mail: ywkwon@snu.ac.kr

**Table of Contents**

| **Supplementary methods** | **p. 4** |
| --- | --- |
| **Supplementary Table 1.** Primer sequences for the amplification of mitochondrial DNA | **p. 6** |
| **Supplementary Table 2.** Primer sequences for quantitative real-time PCR | **p. 7** |
| **Supplementary Figure 1.** Experimental design of in vitro and in vivo models. | **p. 8** |
| **Supplementary Figure 2.** Selective hepatic engraftment of ESC-MSCs. | **p. 9** |
| **Supplementary Figure 3.** Hepatic inflammatory cytokine expression following ESC-MSC transplantation in CDAHFD-fed mice. | **p. 11** |
| **Supplementary Figure 4.** ESC-MSC–derived HGF directly activates AMPK signaling. | **p. 12** |
| **Supplementary Figure 5.** Proposed mechanism of ESC-MSC–mediated metabolic improvement. | **p. 14** |

**Supplementary Methods**

***Measurement of hepatocyte growth factor (HGF) in cell culture supernatants and murine liver tissue***

HGF levels in embryonic stem cell-derived mesenchymal stem cell (ESC-MSC) culture supernatants and mouse liver tissue lysates were quantified using the Human HGF SimpleStep ELISA® Kit (Abcam, Cambridge, UK), following the manufacturer’s instructions. Cell-free supernatants collected after indirect co-culture and cleared liver tissue homogenates were analyzed by measuring absorbance at 450 nm with a microplate reader. HGF concentrations were calculated from a standard curve and expressed as pg/mL.

***HGF treatment assay***

To determine whether HGF directly activates AMP-activated protein kinase (AMPK) signaling, recombinant human HGF (50 ng/mL; R&D Systems, Minneapolis, MN, USA) was applied to palmitic acid-treated HepG2 cells for 3 hours. Expression levels of phosphorylated AMPK and peroxisome proliferator-activated receptor gamma coactivator 1-alpha were analyzed by Western blot.

***Histological analysis and assessment of ESC-MSC engraftment***

Liver tissues were fixed in 10% formaldehyde, embedded in paraffin, sectioned at 5 µm thickness, and stained with hematoxylin and eosin for histological analysis using a light microscope (Nikon ECLIPSE Ci-L, Tokyo, Japan). For assessment of ESC-MSC engraftment, liver and lung tissues were processed similarly. After deparaffinization (National Diagnostics, Atlanta, GA, USA) and rehydration, antigen retrieval was performed by boiling the sections in Target Retrieval Solution (Dako, Glostrup, Denmark) for 30 minutes. Endogenous peroxidase activity was blocked with 3% hydrogen peroxide for 10 minutes. Sections were permeabilized (0.025% Triton X-100 in PBS) and blocked (PBS containing 10% FBS and 1% BSA) for 1 hour. Samples were incubated overnight at 4 °C with anti–human leukocyte antigen (HLA) antibody (1:2000; Abcam), followed by anti–rabbit SignalStain® Boost Detection Reagent (Cell Signaling Technology, Danvers, MA, USA). Detection was performed using DAB (Dako) and hematoxylin counterstaining. Slides were mounted and evaluated microscopically.

***Genomic DNA extraction and human-specific Alu qPCR assay***

Genomic DNA was extracted from mouse tissues (liver, lung, and spleen) and human ESC-MSCs using the QIAamp DNA Mini Kit (QIAGEN, Hilden, Germany) according to the manufacturer’s protocol. For absolute quantification, a standard curve was generated by serially diluting human ESC-MSC gDNA into control mouse liver gDNA (R^2^=0.996). Data are expressed as human cell equivalents per reaction, with 0.1 cell equivalents defined as the lower limit of quantification.

**Supplementary Tables**

Supplementary Table 1. Primer sequences for the amplification of mitochondrial DNA

| **Gene** | **Sequence** |
| --- | --- |
| Human mtDNA | forward, 5'-CGAAAGGACAAGAGAAATAAGG-3' |
|  | reverse, 5'-CTGTAAAGTTTTAAGTTTTATGCG-3' |
| Mouse mtDNA | forward, 5'-AAATTTCGGTTGGGGTGACCT-3' |
|  | reverse, 5'-CTGGATTGCTCCGGTCTGAA-3' |
| Human β-actin | forward, 5'-AGGATGCAGAAGGAGATCACT-3' |
|  | reverse, 5'-GGGTATAACGCAACTAAGTCATAG-3' |
| Mouse β-globin | forward, 5'-CAGGAGCTGTGGGAGGAAGATAA-3' |
|  | reverse, 5'-TTATGGTTGGGATAAGGCTGGATT-3' |

Abbreviations: mtDNA, mitochondrial DNA.

Supplementary Table 2. Primer sequences for quantitative real-time PCR

| **Gene** | **Sequence** |
| --- | --- |
| Human PGC1α | forward, 5'-TGCCCTGGATTGTTGACATGA-3' |
|  | reverse, 5'-TTTGTCAGGCTGGGGGGTAGG-3' |
| Mouse PGC1α | forward, 5'-AATTGAAGAGCGCCGTGT-3' |
|  | reverse, 5'-AACCATAGCTGTCTCCATC-3' |
| Human PPARα | forward, 5'-CCACCATCGCGACCAGAT-3' |
|  | reverse, 5'-TCCGAGGGCTCTGTCATC-3' |
| Mouse PPARα | forward, 5'-GGGCAGCTGACTGAGGAA-3' |
|  | reverse, 5'-GACGTGCTTCCTGCTTCATAGA-3' |
| Human β-actin | forward, 5'-AGGATGCAGAAGGAGATCACT-3' |
|  | reverse, 5'-GGGTGTAACGCAACTAAGTCATAG-3' |
| Mouse 18S rRNA | forward, 5'-GTAACCCGTTGAACCCCATT-3' |
|  | reverse, 5'-CCATCCAATCGGTAGTAGCG-3' |

Abbreviations: AMPK, AMP-activated protein kinase; PCR, polymerase chain reaction, PGC1α, peroxisome proliferator-activated receptor gamma coactivator 1-alpha; PPARα, peroxisome proliferator-activated receptor alpha.

**Supplementary Figures**

**Supplementary Figure 1.** Experimental design of in vitro and in vivo models. (A) Schematic of PA-induced HepG2 cell model and ESC-MSC co-culture setup. (B) Experimental timeline of CDAHFD-fed mice and ESC-MSC intravenous injection. Abbreviations: CDAHFD, choline-deficient, L-amino acid-defined, high-fat diet; ESC-MSC, embryonic stem cell-derived mesenchymal stem cell; PA, palmitic acid.

**Supplementary Figure 2.** Selective hepatic engraftment of ESC-MSCs. Representative immunohistochemical staining for human HLA showing positive ESC-MSC engraftment in the liver (A) but not in the lung (B) of CDAHFD + ESC-MSC mice. No positive staining was observed in control or CDAHFD + vehicle groups (serving as negative controls to confirm antibody specificity without non-specific binding), confirming selective hepatic localization of transplanted ESC-MSCs. (C) Quantitative assessment of human ESC-MSC-derived genomic DNA in liver, lung, and spleen tissues by human-specific Alu quantitative real-time PCR (qPCR). Data are expressed as mean ± SD of human cell equivalents per reaction. The horizontal dotted line denotes the validated lower limit of quantification (0.1 cell equivalents/reaction). Abbreviations: CDAHFD, choline-deficient, L-amino acid-defined, high-fat diet; ESC-MSC, embryonic stem cell-derived mesenchymal stem cell; HLA, human leukocyte antigen; LLOQ, lower limit of quantification; qPCR, quantitative real-time polymerase chain reaction.

**Supplementary Figure 3.** Hepatic inflammatory cytokine expression following ESC-MSC transplantation in CDAHFD-fed mice.

Quantitative RT-PCR analysis of pro-inflammatory cytokine mRNA expression in liver tissues from Control, CDAHFD + vehicle, and CDAHFD + ESC-MSC groups: (A) relative *Ifng* (IFN-γ) mRNA levels, (B) relative *Tnf* (TNF) mRNA levels, and (C) relative *Il6* (IL-6) mRNA levels. Data are presented as mean ± SD normalized to the control group; Group differences were evaluated by one-way ANOVA followed by Tukey’s post-hoc test; **P*< 0.05, ***P*< 0.01. Abbreviations: CDAHFD, choline-deficient, L-amino acid-defined, high-fat diet; ESC-MSC, embryonic stem cell-derived mesenchymal stem cell; IFN-γ, interferon gamma; IL-6, interleukin 6; mRNA, messenger ribonucleic acid; RT-qPCR, reverse transcription-quantitative polymerase chain reaction; SD, standard deviation; TNF, tumor necrosis factor.

**Supplementary Figure 4.** ESC-MSC–derived HGF directly activates AMPK signaling.

(A) Quantification of HGF levels in co-culture supernatants by ELISA. (B) Quantification of human HGF concentrations in mouse liver lysates by human-specific ELISA. The dotted horizontal line indicates the lower limit of quantification (78.13 pg/mL). (C–D) Western blot analysis showing increased phosphorylation of AMPK and upregulation of PGC1α after recombinant HGF treatment in PA-treated HepG2 cells. Group differences were evaluated by one-way ANOVA followed by Tukey’s post-hoc test; **P*< 0.05, ***P*< 0.01. Abbreviations: AMPK, AMP-activated protein kinase; ELISA, enzyme-linked immunosorbent assay; ESC-MSC, embryonic stem cell-derived mesenchymal stem cell; HGF, hepatocyte growth factor; PA, palmitic acid; PGC1α, peroxisome proliferator-activated receptor gamma coactivator 1-alpha.

**Supplementary Figure 5.** Proposed mechanism of ESC-MSC–mediated metabolic improvement. Schematic summary illustrating that ESC-MSCs secrete HGF, which acts in a paracrine manner on recipient hepatocytes to activate the AMPK–PGC1α–PPARα axis, leading to restoration of mitochondrial oxidative function and amelioration of hepatic steatosis in MASLD. Abbreviations: AMPK, AMP-activated protein kinase; ESC-MSC, embryonic stem cell-derived mesenchymal stem cell; HGF, hepatocyte growth factor; MASLD, metabolic dysfunction-associated steatotic liver disease; PGC1α, peroxisome proliferator-activated receptor gamma coactivator 1-alpha; PPARα, peroxisome proliferator-activated receptor alpha.
